# A Low-Power 12-Bit 20 MS/s Asynchronously Controlled SAR ADC for WAVE ITS Sensor Based Applications

**DOI:** 10.3390/s21072260

**Published:** 2021-03-24

**Authors:** Khuram Shehzad, Deeksha Verma, Danial Khan, Qurat Ul Ain, Muhammad Basim, Sung Jin Kim, Behnam Samadpoor Rikan, Young Gun Pu, Keum Cheol Hwang, Youngoo Yang, Kang-Yoon Lee

**Affiliations:** Department of Electrical and Computer Engineering, Sungkyunkwan University, Suwon 16419, Korea; khuram1698@skku.edu (K.S.); deeksha27@skku.edu (D.V.); danialkhan@skku.edu (D.K.); quratulain@skku.edu (Q.U.A.); basim@skku.edu (M.B.); sun107ksj@skku.edu (S.J.K.); behnam@skku.edu (B.S.R.); hara1015@skku.edu (Y.G.P.); khwang@skku.edu (K.C.H.); yang09@skku.edu (Y.Y.)

**Keywords:** asynchronous control logic, successive approximation register (SAR), wireless access in vehicular environments (WAVE), low power consumption, capacitive digital to analog converter (CDAC)

## Abstract

A low power 12-bit, 20 MS/s asynchronously controlled successive approximation register (SAR) analog-to-digital converter (ADC) to be used in wireless access for vehicular environment (WAVE) intelligent transportation system (ITS) sensor based application is presented in this paper. To optimize the architecture with respect to power consumption and performance, several techniques are proposed. A switching method which employs the common mode charge recovery (CMCR) switching process is presented for capacitive digital-to-analog converter (CDAC) part to lower the switching energy. The switching technique proposed in our work consumes 56.3% less energy in comparison with conventional CMCR switching method. For high speed operation with low power consumption and to overcome the kick back issue in the comparator part, a mutated dynamic-latch comparator with cascode is implemented. In addition, to optimize the flexibility relating to the performance of logic part, an asynchronous topology is employed. The structure is fabricated in 65 nm CMOS process technology with an active area of 0.14 mm^2^. With a sampling frequency of 20 MS/s, the proposed architecture attains signal-to-noise distortion ratio (SNDR) of 65.44 dB at Nyquist frequency while consuming only 472.2 µW with 1 V power supply.

## 1. Introduction

Internet of Things (IoT) is considered as a challenging technology and next growth engine that will have an everlasting effect in the semiconductor field. IoT devices have capability to connect a plenty of different end systems. IoT based techniques applied on traffic management systems result in an intelligent and advanced transportation system. A wireless access in vehicular environments (WAVE) is a protocol related to vehicle communications and provides an efficient, and reliable radio communications in an intelligent transportation system (ITS). In most of the ITS application, the WAVE protocol system has been designed in such a way to allow one vehicle to communicate with other vehicles (V2V), to other device (V2R), or to infrastructures (V2I) via dedicated short-range communications (DSRC) [1,2]. WAVE protocol has the potential to carry out an authentic and competent V2V, V2I and V2R communications to facilitate the mobility, safety and environmental applications. It consists of an on-board equipment (OBE) and a roadside equipment (RSE), wirelessly connected to provide an intelligent system. An on-board equipment (OBE) generally should offer low power, low cost, low design complexity, good reliability, and high energy efficiency. A fully-integrated RF-SoC is a most suitable option to meet the above mentioned requirements.

**Figure 1:**
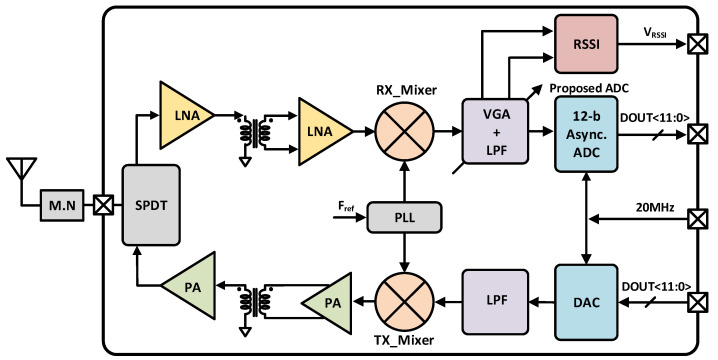
Top architecture of the DSRC transceiver.

Figure 1 plots the top block diagram of highly integrated 5.8 GHz DSRC transceiver system. It satisfies the aforementioned requirements without any externally connected block like low-noise amplifier (LNA) and external power amplifier (PA) [3]. The main building blocks consists of a matching network (M.N), single pole double throw (SPDT) switch, an inductively generated low-noise amplifier (LNA) to amplify the input signal, a mixer (MIXER), a 12-bit ADC, a 12-bit DAC, a received signal strength indicator (RSSI), a variable gain amplifier (VGA) with a low pass filter (LPF) and power amplifier (PA) [4]. An integrated SAR ADC allows transceiver to communicate with the digital baseband [5].

As a result of the breakneck advancement in wireless technologies, a number of communication standard applications including ITS transceivers require on-chip ADCs with a sampling speed of few tens of MS/s and a resolution of more than 10 bits. The converters for finest communication systems, which include wireless local area networks (WLANs) based on IEEE standards protocol IEEE 802.11 require comparatively higher resolution of more than 10-bit and sampling rate of about few tens of MS/s [6].

Successive approximation register (SAR) analog-to-digital converters (ADCs) have been proven to be energy efficient in achieving moderate resolution and speed range [7,8] having a single comparator structure with no static power consumption and comparatively a simple structure. Recently, SAR algorithm based ADCs have also been used for higher speed and medium resolution applications by time interleaving multiple sub-SAR channels replacing traditionally implemented flash or pipeline structures [9]. However, with the increased number of bits, limitations due to comparator noise become severe which make SAR ADC as a difficult approach to implement for high resolution [10,11]. An energy-efficient prototype for high resolution is implemented front-end sampling switch, which results in eliminating the timing skew [12]. For high resolution ADCs, capacitive DAC consumes very high switching energy [13], for noise filtering integrator-based amplifier is used in [14]. Re-configurability and bandwidth scalability is achieved in [15] SAR ADC at a cost of comparatively high power consumption. A top-plate sampling increases the precision for 12-bit due to the implemented bootstrap switch. For a smaller overall capacitance, a DAC configurable binary window switching technique is implemented in [16]. However, it is lagging behind in terms of energy efficiency. For fully differential architecture, several techniques have been implemented to decrease the capacitor array size without digital calibration [17]. To reduce the switching energy and improve the linearity, floating DAC switching technique is presented in [18]. In [19,20], a binary-window DAC switching technique is presented to decrease switching error and DAC non-linearity at the cost of excessive power consumption. To decrease the distortion introduced by threshold voltage and parasitic capacitance, a linearity enhancement switch is implemented in [21]. A top-plate sampling technique is used to reduce the capacitor array size by half in [22], but it can cause non-linearity and common mode dependency upon input. A bottom plate sampling method is presented in [23] to reduce the overall size of the capacitor array.

This paper presents a 12-bit, 20 MS/s asynchronously controlled fully differential SAR ADC for wide-band WAVE based DSRC transceiver systems. To improve the static and dynamic performance of ADC, various techniques have been implemented. For 12-bit ADC, the implemented switching technique with CMCR switching conversion reduces the switching energy of DAC by 56.3% as compared to the conventional CMCR switching technique. The top-plate sampling results in increased settling because the influence of charge injection is reduced due to the aligned switching (AS) and detect-and-skip (DAS) switching technique. The implemented bootstrap switching technique improves the static performance of ADC. A constant DC shift and gain error can be introduced by the sustainable charge injection error, and sampling linearity will not deteriorate by implemented bootstrap switches. To decrease the power consumption and kickback noise of ADC, the proposed dynamic latch comparator with cascode is used.

The top configuration of the proposed asynchronous SAR ADC architecture is depicted in Section 2. The sub-blocks of the proposed ADC, such as the proposed capacitive DAC with a modified CMCR switching method, bootstrap switching, and dynamic latch comparator with cascode are explained in Section 3. The measured results and the performance summary of the presented ADC architecture is discussed in Section 4, and finally, we conclude our brief in Section 5.

## 2. The Top-Block Diagram of Proposed ADC Architecture

**Figure 2:**
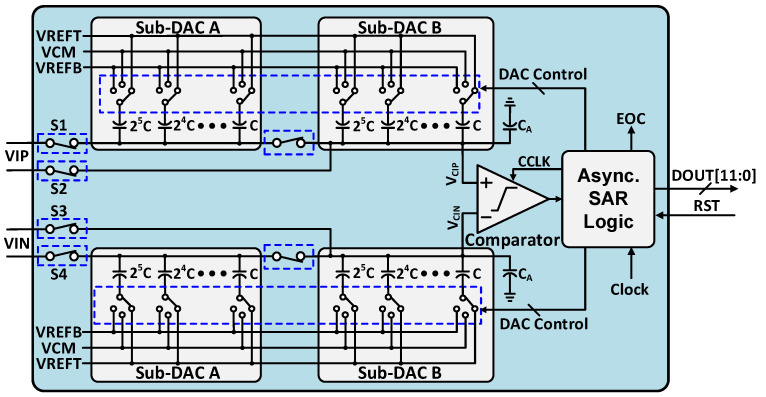
Top block diagram of proposed 12-bit asynchronous SAR ADC.

The presented configuration of SAR ADC is depicted in Figure 2. The presented architecture contains a comparator, SAR logic, clock generator, binary-weighted capacitive DAC and bootstrap switch. To improve the common-mode noise rejection and to reduce the noise of supply voltage we have implemented a fully differential architecture of SAR ADC. By bootstrap switches, the differential input signal is sampled at the bottom plate of capacitive DAC. According to the comparator decision and output digital code stored by the modified asynchronously controlled SAR logic, which controls the capacitive DAC switches.

## 3. Circuit Implementation

### 3.1. Capacitive DAC with Modified CMCR Switching Technique

**Figure 3:**
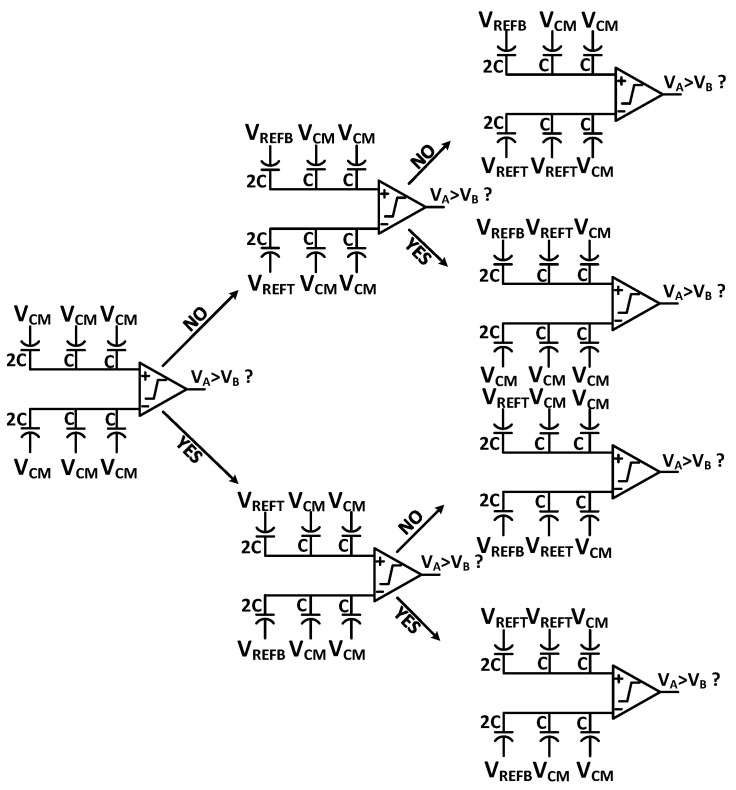
A 3-bit example of CMCR switching scheme.

In a conventional switching scheme, for N-bit resolution, SAR ADC usually requires 2^N^ number of unit capacitors. The number of the unit capacitor can be reduced by optimizing the capacitive DAC’s switching sequence, which is broadly explored, such as common mode based switching, set-and-down [24,25], and so on. The Area and power consumption of the capacitive DAC are significantly large for the high-resolution ADC such as over 10-bit resolution. To lower the capacitance from the DAC part, we adopt the common-mode charge recovery (CMCR) switching method [26]. With this switching, we use the possible minimum size of the unit capacitor in a capacitive DAC layout. An example of a 3-bit CMCR switching sequence is represented in Figure 3.

**Figure 4:**
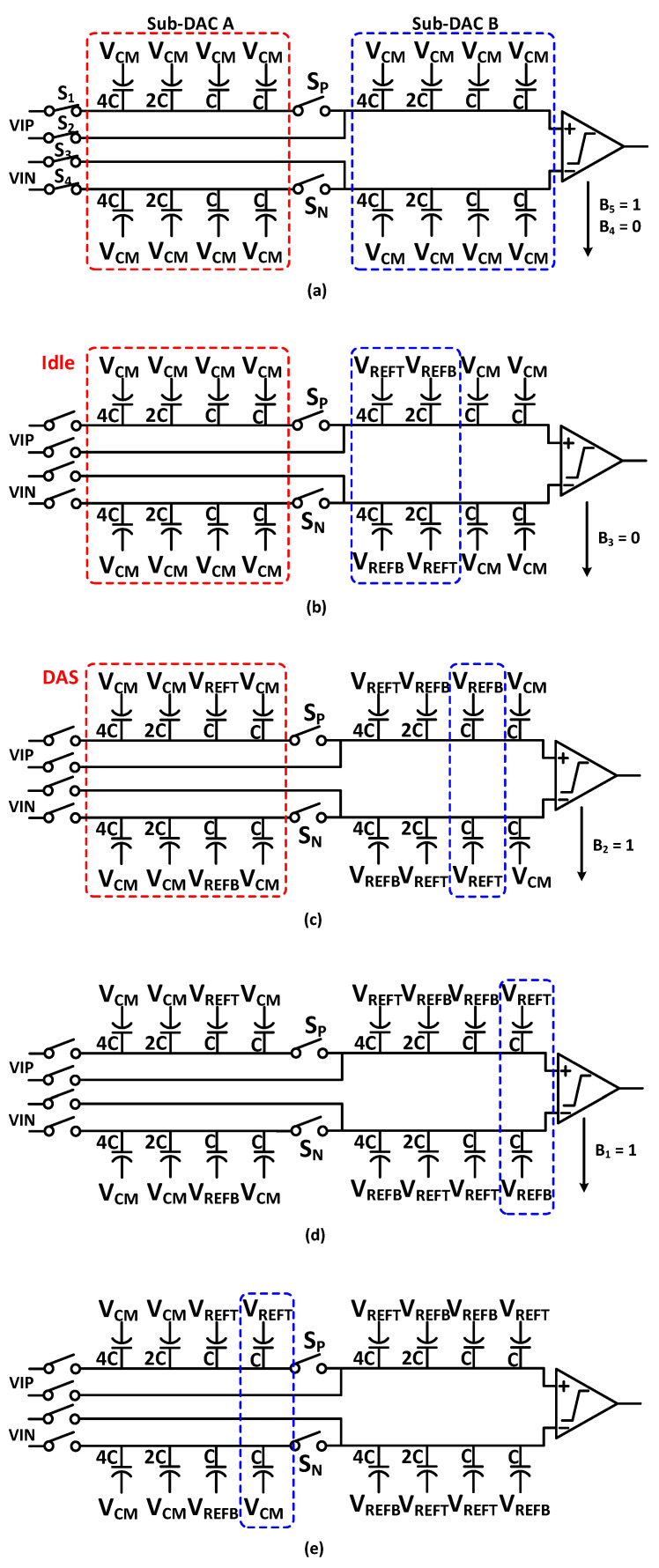
Example of proposed switching technique for 6-bit SAR architecture (**a**) Sampling phase (**b**) First two conversion cycles (**c**) 3rd conversion cycles (**d**) 4th conversion cycle (**e**) 5th conversion cycle.

The CMCR switching technique operation is based on the common-mode voltage scheme anticipated by the last comparison cycle. An additional bit is monotonously converted to the lowest capacitor of capacitive DAC to V_REFT_ from V_CM_. Due to differential implementation, noise can be eliminated by the CMCR switching technique, furthermore, this switching method introduced the ripple in the LSB conversion by V_CM_. Although, VCM can easily implement 1-bit accuracy, and it is effective for the reduction of the cost of sampling switches and DAC. We propose a switching technique based on the CMCR switching method for 12-bit SAR ADC as shown in Figure 4. Switches S_1_~S_4_ are input sampling switches which sample the input signal to the sub-DACs. The driving requirement of the SAR logic and comparator must be satisfied by the DAC control switches. For the 12-bit ADC, large switches are needed to achieve the charge sharing within the restricted time, because the peak-to-peak value of voltage discrepancy is V_REF_ on the top plate of the capacitive DAC, which causes the large power dissipation. Besides, the loss of switching energy in the proposed switching technique for the first ten comparison cycle is 2/4 times in comparison with the 10-bit CMCR switching method for the DAC capacitance increase.

The proposed DAC switching sequence use the detect-and-skip (DAS) technique, and aligned switching (AS) technique to minimize the switching energy loss and the size of DAC controlling switches S_1_~S_4_, in the comparison phase [14]. The proposed switching procedure prosecute with two steps:

(1) LSB conversion by the whole sub-DACs.
(2) MSB conversion by one sub-DAC.

**Figure 5:**
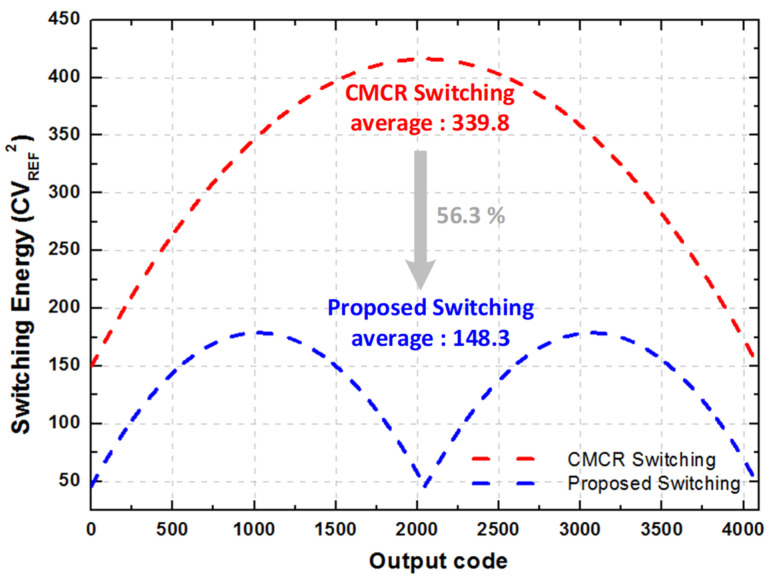
Comparison between switching energies of the proposed and conventional switching technique.

**Figure 6:**
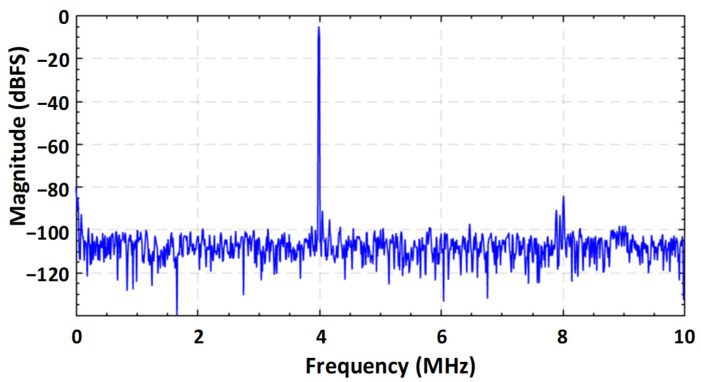
Dynamic performance with behavioral model of proposed switching technique with 1% unit capacitor mismatch.

**Figure 7:**
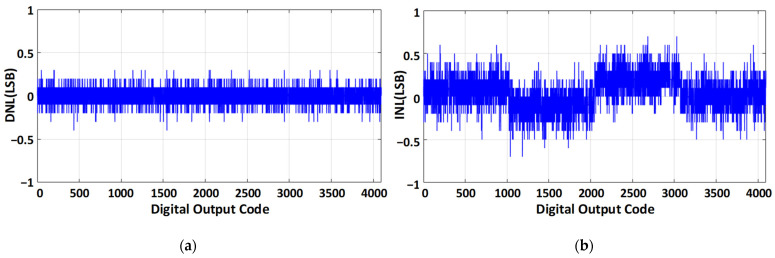
Static performance with behavioral model of proposed switching technique with 1% unit capacitor mismatch (**a**) differential non-linearity (DNL), and (**b**) integral non-linearity (INL).

The proposed switching technique for 6-bit SAR ADC which contains two sub-DACs is represented in Figure 4. In the implemented DAC, with the CMCR switching technique ADC start the conversion of sampling signal after the sampling phase, while sub-DAC A does not consume any switching energy because it is idle. By using the DAS and AS technique, the data transferred to sub-DAC A after the generation of the first three bits (B3~B5). Simultaneously, B2 determines by the comparator. When the sub-DAC B’s LSB capacitors are switched by B2, the switches S_P_ and S_N_ are switched on in the 4th comparison cycle. During the 3rd comparison cycle, AS sets up and sub-DACs switching does not require any additional settling time. In the end, by the CMCR switching technique, the bits (B1~B0) are converted. By the proposed switching method, we are able to reduce the loss of switching energy from the capacitive DAC part, because the generation of the first nine bits is done by only one sub–DAC, and others are idle. The comparison between the conventional switching and the proposed switching energy versus output code is shown in Figure 5. The proposed switching method consumes 56.3% less energy when compared to the conventional CMCR switching method. The voltage variation is very small at the top plate, and DAC controlling switches S_1_~S_4_ requirements are reduced because they turn on after the achievement of nine bits. The switching energy is very efficient for the DAS and AS techniques. The dynamic performance with behavioral simulation is done in MATLAB^®^ of proposed switching technique with 1% unit capacitor mismatch is shown in Figure 6. The static performance differential non-linearity (DNL), and integral non-linearity (INL) behavioral model of proposed switching technique with 1% unit capacitor mismatch is shown in Figure 7.

For 12-bit, unit capacitor size is calculated with 1 V power supply by considering the capacitor mismatch and thermal noise power from DAC and due to sampling. The effective noise power due to sampling and from DAC is calculated by following the Equations (1) and (2) respectively,

(1) $$v_{ns}^{2}=\frac{2KT}{C_{SAM}}$$

(2) $$v_{nd}^{2}=\frac{2KT\left(C_{DAC}\right)}{\left(C_{SAM}\right)\left(C_{A}\right)}$$

where, $v_{ns}^{2}$ is noise because of sampling,$v_{nd}^{2}$ is effective noise from DAC, *K* is Boltzman’s constant, *T* is the temperature, *C_SAM_* is the total sampling capacitance, *C_A_* is the intentional grounding capacitor for attenuation, and *C_DAC_* is the overall DAC capacitance. The total sampling capacitance is 286 C, where C is the unit capacitance with a value of 15 fF.

### 3.2. Bootstrap Switch

**Figure 8:**
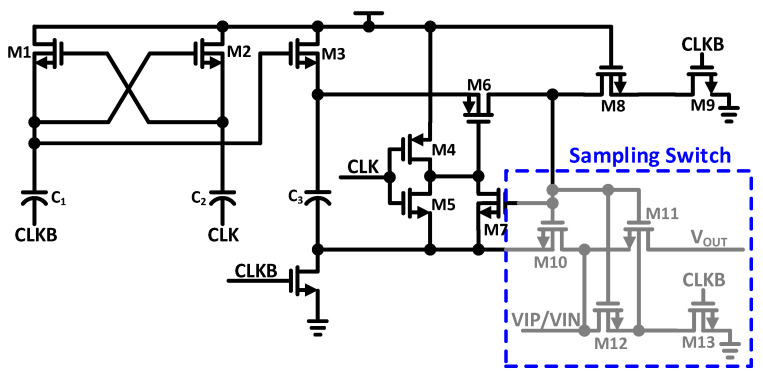
Bootstrap Switching Schematic.

Figure 8 shows the employed schematic of bootstrap switching, which is improved as proposed in [27]. The implemented bootstrap switch operates at the supply voltage. The gate body voltage (V_GB_) of transistor M11 will be twice the supply voltage. Deep N-well (DNW) transistors M10–M13 are used to reduce the risk of enhancing reliability and failure. Transistors M10–M12 are turned on during the sampling phase. During this phase, the gate source voltage (V_GS_) and V_GB_ of transistor M11 abide to supply voltage. During the sampling period, the implemented procedure is also competent to increase the sampling linearity and abolish the body effect because the body source voltage (V_BS_) of transistor M11 stays at zero. When the substrate of transistor M11 goes to zero, while M13 is turned on then transistors M10–M12 are turned off during the conversion phase. In this way, we can ensure the separation of input V_IP_/V_IN_ from the output V_OUT_, because both drain substrate pn junction and source substrate pn junction are inversely-biased.

For the differential architecture, we assume that the bootstrap switches matching accuracy is sufficient, since small common mode variation is caused by this, and the clock feed-through effect can be ignored. By embracing the implemented bootstrap switch architecture, we can alleviate the body-effect impact. On the differential inputs, a constant DC shift and gain error can be introduced by the sustainable charge injection error, and sampling linearity will not deteriorate. Hence, the clock feed-through and the charge injection’s negative effect is attenuated by the differential architecture.

### 3.3. Dynamic Latched Comparator

To decrease the power consumption of ADCs, dynamic latched comparators are frequently used [28]. Several issues have been considered during the comparator designing such as; due to the comparator’s clock operation, kickback noise affects the CDAC top plate. During the monotonic switching, offset voltage V_offset_ dependent on the V_CM_ and V_offset_ generated by device mismatch. The clock transition of the comparator distributes the comparator differential input V_CIP_ and V_CIN_. In the proposed dynamic latched comparator, when the comparator clock signal CCLK goes to high then the input difference is settled. By the clock feed-through, at the comparator input, kickback noise is created during the CCLK transition. When the comparator input V_CIP_ and V_CIN_ sort out to a stable voltage then there is a recovery period. The comparator begins to sort out the variance between inputs, during this recovery period. Decision error can cause by a small asymmetry in the recovery period.

**Figure 9:**
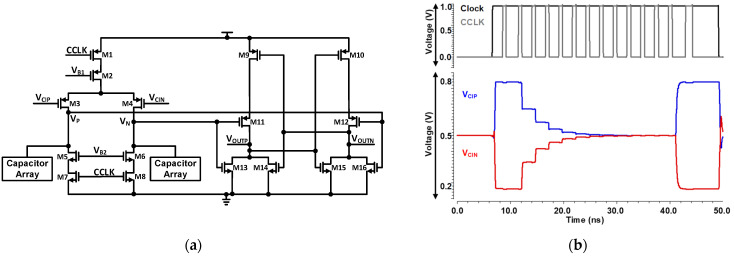
(**a**) Schematic of Dynamic Latched Comparator using Cascode to reduce kick-back (DNL), and (**b**) Waveform represented the CDAC settling with comparator.

The implemented architecture of the comparator is depicted in Figure 9a. Due to the process variation, mismatch and hysteresis can exist in the comparator because of the use of transistors M13 and M16. Therefore, to minimize the hysteresis common centroid layout is used. To reduce the kickback noise and common mode dependent offset calibration the proposed comparator is designed. Cascode transistors M2, M5, and M6 shield the input transistors M3 and M4 to reduce the kickback noise of the comparator. The aspect ratio of cascode transistors M2, M5, and M6 is small so that these transistors operate in the saturation region and increase the output resistance of these transistors. Increased output resistance attenuates the large voltage step produced in result of the transition from CCLK. By the circuit simulation, we choose the bias voltages V_B1_ and V_B2_ and size of the cascode transistors. We control the current of transistors M5 and M6 through bias voltage V_B2_. During the CCLK transition, the peak current reduced through transistors M5 and M6 when bias voltage V_B2_ is reduced. Furthermore, we optimize the size of input transistors M3 and M4. The kickback peak value is reduced by using the size optimization and cascode transistors. Figure 9b represents the DAC output voltages V_CIP_ and V_CIN_. When CCLK goes to high from low, the input difference is minimized in the dynamic latched comparator. At the comparators input, kickback noise is generated by clock feed-through of CCLK transition. When the comparators input V_CIP_ and V_CIN_ settles to a stable voltage, then there is a recovery period and in this time period input difference of comparator start to resolve, decision error can cause by a small asymmetry in the recovery period.

Comparator offset calibration is performed by using binary-weighted capacitor array to control voltage offset *V_offset_*. We select digital approach instead of analog offset calibration, because it requires additional DAC [29]. The voltage offset *V_offset_* consists the dynamic and static offset of the comparator. *V_offset_* of comparator can be derived as:

(3) $$V_{offset}=\Delta V_{TH3,4}+\frac{V_{SG}-\left|V_{TH3,4}\right|}{2}\left(\frac{\Delta {\left(W/L\right)}_{3,4}}{{\left(W/L\right)}_{3,4}}+\frac{\Delta R_{load}}{R_{load}}\right)$$

where *V_TH3,4_* is the threshold voltage, ∆*V_TH3,4_* is the threshold mismatch, ∆*R_load_* is the load resistance mismatch, and ∆*(W/L)_3,4_* is the physical dimension mismatch between transistors M3 and M4.

### 3.4. Asynchronous SAR Logic Processing

**Figure 10:**
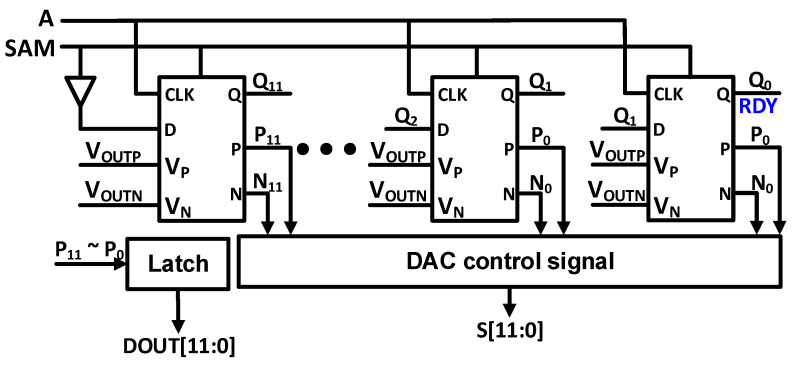
SAR logic block diagram.

Asynchronous SAR control logic avoids the need of high frequency external clock signal as all conversions are carried out in a single clock cycle. To optimize the flexibility relating to the performance of logic part, an asynchronous topology is employed as shown in Figure 10.

**Figure 11:**
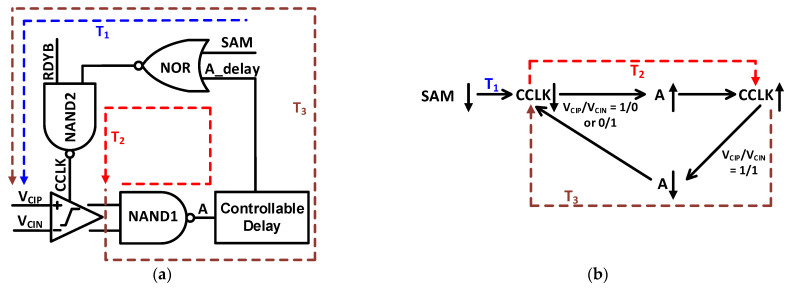
Asynchronous CCLK generator (**a**) Schematic of asynchronous clock generator with controllable delay (**b**) Logic description of CCLK generation.

To optimize the DAC switching and conversion time, we have added the digitally controllable delay cells for each conversion. Schematic and logic explanation of modified asynchronous clock generator CCLK is presented in Figure 11. The comparator output reset to VDD, when CCLK signal is high which is controlled by sampling signal SAM. SAM is the modified Clock signal with changed duty cycle. After sampling phase SAM signal goes to low and CCLK makes the comparator starts working after T_1_ time. The comparator’s outputs generate a high signal A through NAND1. After T_2_ time, CCLK goes to high which results in resetting the comparator for further comparison. After T_3_ time, the comparator’s outputs goes to high which generates a low signal A and comparator is triggered. Digitally controllable delay cell is added in time T_3_, to optimize the conversion speed, depending upon the settling time of the DAC.

## 4. Measurement Results

**Figure 12:**
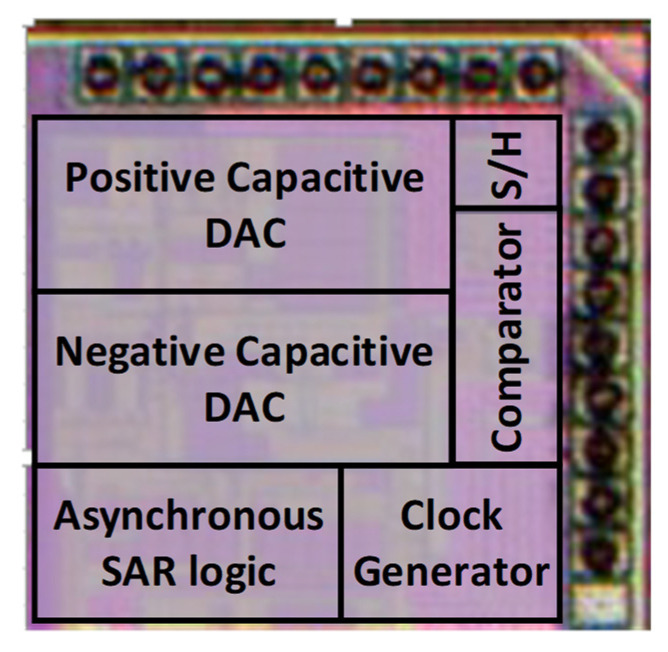
Die photograph of the implemented ADC.

**Figure 13:**
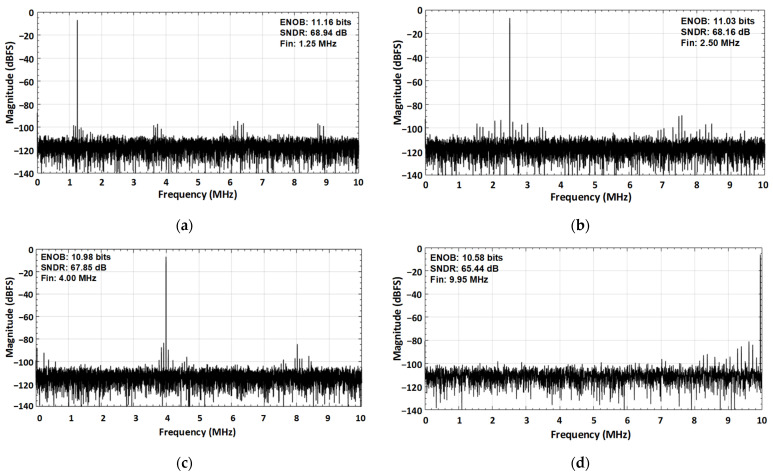
Measured dynamic performance for two different input frequencies at the sampling rate of 20 MS/s (**a**) for 1.25 MHz input frequency (**b**) for 2.50 MHz input frequency (**c**) for 4.00 MHz input frequency, and (**d**) for nyquist input frequency of 9.95 MHz.

In a one poly six metal (1P6M) 65 nm CMOS technology, the fabricated prototype occupies an active chip area of 0.14 mm^2^ as shown if Figure 12. The marked contents of the die micrograph correspond to each sub block of the proposed SAR ADC. The measured dynamic performance of the proposed ADC at two different frequencies is shown in Figure 13. The FFT spectrum shows that it achieves an ENOB of 10.98 bit at 4 MHz input frequency and 10.58 bit at around Nyquist input frequency with a sampling rate of 20 MS/s and an input single with a peak-to-peak voltage range of 600 mV.

**Figure 14:**
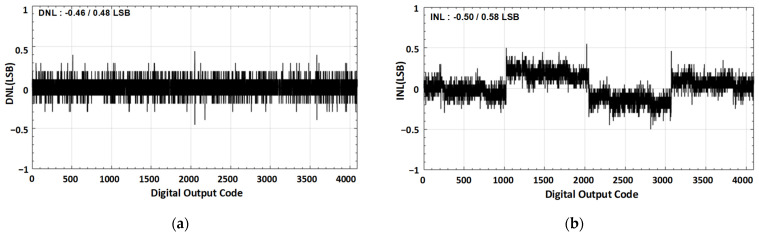
Static performance parameter (**a**) DNL, and (**b**) INL.

Figure 14 presents the measured static performance. The peak differential non-linearity (DNL) and integral non-linearity (INL) values are +0.6/−0.6 LSB and +0.9/−0.9 LSB, respectively.

**Figure 15:**
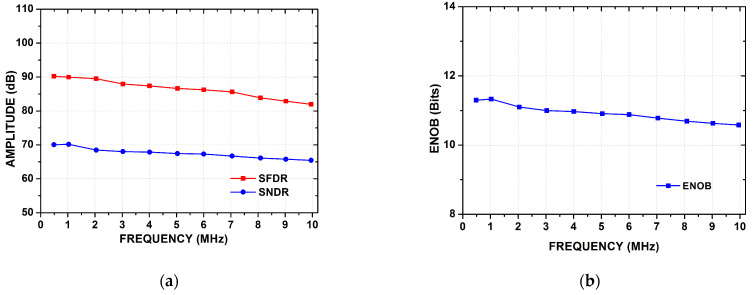
(**a**) Input frequency versus SNDR and SFDR. (**b**) Input frequency versus ENOB.

**Figure 16:**
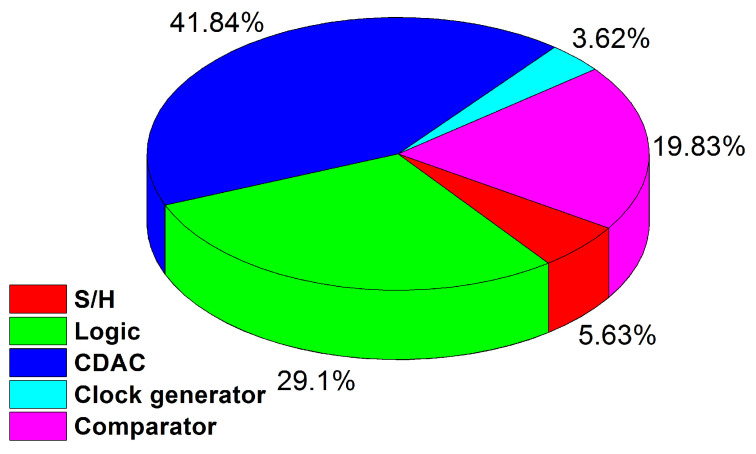
Breakdown of power consumption of ADC.

Figure 15a presents the trend of SFDR and SNDR versus the applied input signal frequencies at 20 MS/s with 1 V of power supply. ENOB variation with respect to input signal frequency is shown in Figure 15b. The breakdown of power consumption with respect of sub blocks is presented in Figure 16.

Table 1 presents the performance summary of the proposed architecture and its comparison with the other state of the art architectures [15,16,19,20,21]. It is evident that the proposed architecture exhibits a competitive performance in terms of energy efficiency and linearity. To evaluate the overall performance of the proposed ADC, the commonly used parameter, Figure of Merit (FOM), is used as

(4) $$FOM\;=\;\frac{P_{ADC}}{\min\left\{F_{S},2\times ERBW\right\}2^{ENOB}}$$

where, *F_S_* denotes the sampling rate and *P_ADC_* is the power consumed by the structure. The proposed structure achieves a *FOM* of 15.42 fJ/conv. step.

## 5. Conclusions

A low power 12-bit, 20 MS/s asynchronously controlled SAR ADC was fabricated with one poly six metal (1P6M) 65 nm CMOS technology to be used in WAVE protocol based intelligent transportation system. Several techniques have been proposed to optimize the architecture with respect to power consumption and performance. To alleviate the switching energy problem of the DAC part, the proposed switching method which employs CMCR switching technique is implemented in CDAC part. A mutated dynamic latch comparator with cascode is implemented to make certain a high speed operation with low power consumption and to overcome the kick back issue. Moreover, the presented modified asynchronous topology in control logic part optimizes the flexibility relating to the performance of logic part. The structure have an active area of 0.14 mm^2^. The presented SAR ADC was operated at a sampling rate of 20 MS/s, attaining a peak SNDR level of 65.44 dB with a peak ENOB of 10.58 bits at Nyquist frequency. While consuming only 472.2 µW of power with 1 V power supply, the proposed architecture achieved a FOM of 15.42 fJ/conv. step.

## Figures and Tables

**Table 1:** Performance summary and comparison.

Parameter	[15]	[16]	[19]	[20]	[21]	This Work
Process (nm)	180	180	180	180	180	65
Supply Voltage (V)	1.8	1.5	1.5	1.2	1.8	1
Resolution (bit)	12	12	12	12	12	12
Sampling Rate (MS/s)	20	20	10	40	10	20
SNDR (dB)	64.6	59.1	63.8	62.5	66.9	65.44
ENOB (bits)	10.44	9.52	10.31	10.09	10.82	10.58
DNL (LSB)	−0.51/0.445	−0.65/0.58	1.05	2.33	0.69	−0.46/0.48
INL (LSB)	−1.01/0.98	−1.06/1.04	1.38	3.1	1.15	−0.50/0.58
Power Consumption (µW)	1770	1220	600	1320	820	472.2
FOM (fJ/conv. step)	63.7	83	47.2	30.4	44.2	15.42

## Data Availability

Not applicable.
